# Investigating conditioned pain modulation in horses: can the lip-twitch be used as a conditioning stimulus?

**DOI:** 10.3389/fpain.2024.1463688

**Published:** 2024-10-24

**Authors:** Severin Blum, Jana Gisler, Emanuela Dalla Costa, Stéphane Montavon, Claudia Spadavecchia

**Affiliations:** ^1^ Anaesthesiology and Pain Therapy Section, Department of Clinical Veterinary Medicine, Vetsuisse Faculty, University of Bern, Bern, Switzerland; ^2^ Veterinary Department of the Swiss Armed Forces, Bern, Switzerland; ^3^ Department of Veterinary Medicine and Animal Sciences, University of Milan, Lodi, Italy

**Keywords:** horse, conditioned pain modulation, thermal threshold, nociceptive withdrawal reflex, pressure pain threshold

## Abstract

Study objective was to evaluate whether the application of a lip twitch could be proposed as conditioning stimulus in the context of a novel Conditioned Pain Modulation (CPM) assessment paradigm for use in horses. The study was a prospective, experimental, randomized trial. Twelve healthy horses were evaluated in two experimental sessions. The lip twitch was used as the conditioning stimulus in both sessions; electrical stimulation was used as the test stimulus in one session, while mechanical and thermal stimulations were used in the other. Differences between thresholds recorded before and during twitching (Δ) as well as their percent (%) change were computed for each stimulation modality as a measure of CPM. Heart rate and respiratory rate were recorded throughout the experiments to monitor physiological reactions, while the general level of stress and aversiveness toward twitching were scored using *ad hoc* behavioural scales. Based on these scores, interruption criteria were defined. Ten and seven horses completed the electrical and mechanical/thermal experimental sessions respectively. For electrical stimulation, median (IQR) Δ was −2.8 (−3.9, −1.1) mA and% change 87.9 (65.7–118.2)%; for mechanical stimulation, Δ was −18.2 (−6.4, −21.4) N and% change 343.5 (140, 365.3)%; for thermal stimulation, Δ was −3.1 (−9.2, −2.1)°C, while% change was not calculated. Heart rate and respiratory rates varied significantly over time, with higher values recorded during twitching. Median stress and aversion scores did not differ between the two sessions. As lip twitching consistently affected thresholds to all stimulation modalities, it can be proposed as effective conditioning method for CPM assessment in horses. The exclusion of subjects due to severe aversion shows that this paradigm cannot be indistinctively applied to all horses and that stringent interruption criteria are necessary to guarantee adequate welfare during testing.

## 1 Introduction

Chronic musculoskeletal pain has a high prevalence in horses as well as in humans. Independently from the originating pathology, it is a frequent cause of poor athletic performance, impaired quality of life and an increasingly perceived welfare concern. The appearance of lameness is often the first recognized clinical sign that a painful process is ongoing and gait assessment in response to diagnostic analgesia is classically used to anatomically localize the source of pain (1–3). This lameness-centered approach justifies why in recent years a multitude of objective tools have been developed to quantify gait asymmetry and to monitor its changes over time (4–6). Furthermore, a large body of novel research has focused on the description of species-specific behavioural indicators of pain, in horses at rest as well as ridden (1, 2, 7–9). On the other side, quantitative sensory testing methods, allowing to define the pain phenotype on a mechanistic base and largely applied in human medicine, have been rather neglected in horses so far, except for algometry (10). Both peripheral and central sensitization phenomena are known to accompany most chronic painful pathologies, as demonstrated for osteoarthritis and laminitis (11). Thus, developing or further refine methodologies to evaluate sensory function and its modulation could be useful to better characterize individual horses affected by chronic pain and to predict response to therapy. In humans, the Conditioned Pain Modulation (CPM) paradigm has been largely applied in research and clinical settings to assess alterations in central pain processing (12). The classical assumption is that in normal conditions pain inhibits pain while, in presence of chronic pain, temporal summation mechanisms are enhanced and endogenous inhibition is reduced, leading to a generalized pain facilitation (13, 14). Even though this assumption has been repeatedly challenged, exploring the phenomenon of Conditioned Pain Modulation in horses appears as an interesting novel opportunity to understand the role of endogenous pain control in this species, in health first and later in presence of chronic pain conditions.

Finding an adequate, reliable, and easy-to-use conditioning noxious stimulus is certainly the first prerequisite for a successful assessment of endogenous pain modulation. Secondly, quantifiable test stimuli which allow to define pain thresholds are necessary, as CPM is calculated as the difference between thresholds measured before and during or just after the application of the conditioning stimulus. Conditioning and test stimuli are typically applied in distant body regions to evoke CPM.

Aim of the present study was to assess whether the application of a common lip twitch as conditioning stimulus would modify the thresholds to electrical, thermal and mechanical stimuli applied to the forelimbs in a consistent fashion. It was hypothesized that: the application of the lip twitch would be able to increase the nociceptive withdrawal reflex (NWR), the pressure pain (PP) and the thermal (T) thresholds to a clinically meaningful extent in healthy horses. If this would be the case, such a paradigm could be considered further to evaluate CPM in horses.

## 2 Materials and methods

### 2.1 Study design

This study was designed as a prospective, experimental, randomized, single cohort trial, which received approval from the Committee for Animal Experimentation of the Canton of Bern, Switzerland (license number BE81/2022). The trial, carried out at the National Equine Center in Bern from November 2022 to January 2023, consisted of two experimental sessions for each subject included. In both sessions, the lip twitch was applied as a conditioning stimulus. As test stimuli, in one session electrical stimulation was used (NWR session) while in the other mechanical and thermal stimulation were used (PP/T session). Nociceptive thresholds were measured before, during and after the application of the lip twitch. For each horse, the sequence of sessions was randomized and at least two weeks elapsed between sessions. The timeline of the experiments is graphically represented in Supplementary Material (Supplementary Figure S1).

### 2.2 Horses

Twelve healthy horses, mare and geldings, older than three years and belonging to the Swiss Armed Forces were included. Horses were kept in single stalls in large stables under standard housing conditions and were regularly ridden or driven. Prior to inclusion in the study, a complete physical examination was performed by two veterinarians (JG, SB) supervised by an experienced equine specialist (SM).

To be included, horses had to be free of clinically detectable orthopaedic, neurologic or systemic diseases, and have no evidence of pain, lameness or mobility impairment. Horses were excluded if they received anti-inflammatory or analgesic drugs in the two weeks prior to the study. Physical and orthopaedic examinations were repeated before each experimental session to ensure that no changes had occurred between appointments. All horses were tested in the afternoon. At least one hour had to elapse between feeding and/or daily training exercise and testing.

### 2.3 Instrumentation and monitoring

Two horses per day took part to the experiment. They were collected from their own stall and brought to a large, empty stable to which they were accustomed to. While one horse was tested in the corridor, fixed by two ropes on each side of the halter, the other was kept in a nearby box with visual contact. Testing equipment was placed on the left side of the horse under testing. Horses were generally very calm when fixed in the corridor, as this was the usual place for being groomed and saddled.

The sites foreseen for ECG electrodes placement, on both sides of the withers and on the left chest, were clipped. Sites for electrical stimulation and NWR recordings electrodes were clipped, defatted, and slightly abraded to obtain a proper impedance.

A telemetric ECG monitoring system (Televet 100, Engel Engineering Services GmbH, Germany) was applied on the horse and fixed with an ad-hoc belt following manufacturer recommendation. Recording was started as soon as the test stimulation equipment was in place and continued until 15 min after twitch removal.

Respiratory rate was visually assessed and recorded every two minutes before and after twitching, and every minute during twitching.

For heart rate and respiratory rate, average values were calculated for the 5 min of baseline recordings preceding test stimulation (baseline), for the 5 min preceding twitch application (baseline stimulation), for the 5 min of twitching (twitch), and for 5 min intervals thereafter up to 15 min after twitch removal. These values were used for statistical analysis.

**Figure 1:**
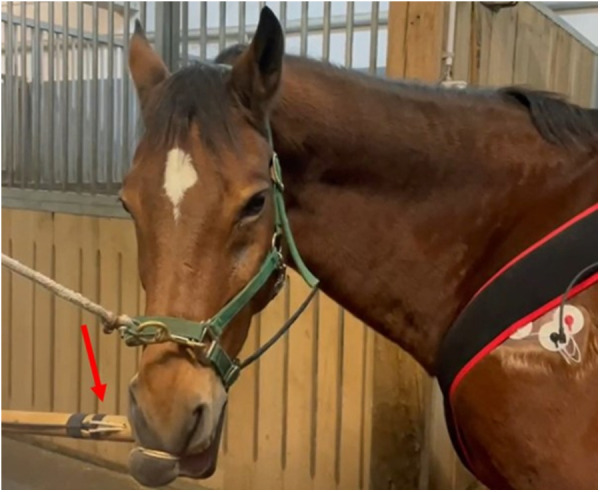
The twitch in place around the horse upper lip. The red arrow shows the position of the force sensor.

**Figure 2:**
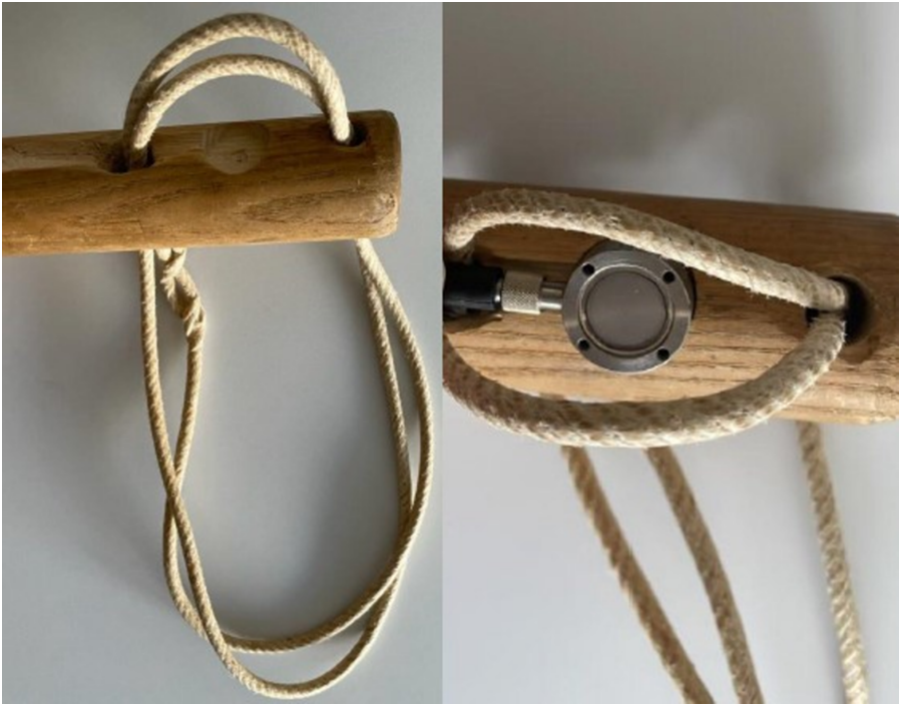
Details of the twitch used in the current trial. On the left, the tip of the wooden handle with the double rope. On the right, the sensor in place under the rope on the back side of the handle.

The twitch, as used for minor procedures in equine veterinary care, consisted in a wooden handle and a double rope to be twisted around the horse's upper lip (Figure 1). During twitching, a round metal sensor (DLM20-BU.500.CP3.M4, Baumer AG, Switzerland) connected to a digital display was placed between the wooden handle and the rope to monitor the force applied (Figure 2). The measured force range was 0–50 N.

### 2.4 Stress and aversion scores

For each experimental session, the level of stress before and after twitching was evaluated every 2 min using a previously validated stress scale, ranging from 1 (no stress) to 10 (high stress) (15).

Furthermore, the degree of aversion shown during twitch application was scored every minute on a scale developed on purpose and ranging from 1 (no aversion) to 4 (severe aversion) (Table 1).

**Table 1:** Scoring system used to evaluate the degree of aversion during twitching. If a stress score >5, or an aversion score >3 was reached, the session was interrupted, and incomplete data sets were discarded. Horses reaching cut-off scores in the first experimental session did not undergo the second experimental session.

Aversion score	Aversion level	Behavioural indicators
1	No	Steady head, eyes open or half closed, ears slowly scanning or still, lips moving freely, tail still or gently swishing. Horse relaxed, calm, accepting, mild signs of sedation possible
2	Mild	Increased head movements, eyes open, increased ears movements or backwards, decreased lips movements. Horse alert, listening, unsettled
3	Moderate	Increased head movements, upward and occasionally against the twitch, pawing/stomping, or freezing with reduced movements but overall increased body tension, eyes open and white showing, tail swishing, defecation. Horse restless, uncomfortable
4	High	Raised head, unsteady and repeatedly moving against the twitch, eyes open and white showing, repeated tail swishing, defecation. Horse agitated, anxious, aggressive behaviour (rearing, barging, pawing against twitch/handler) any time possible or present.

### 2.5 NWR session

In horses undergoing the NWR session, the area over the left palmar digital nerve and the left deltoid muscle were shaved and defatted for the application of surface self-adhesive electrodes (Ambu Blue Sensor N, Ambu Sdn, Malaysia). These electrodes, with an active surface of 0.8 cm^2^, were used for electrical stimulation and NWR recordings, respectively.

For the determination of the NWR threshold, a continuous threshold tracking device (Paintracker V1, Dolosys GmbH, Germany) was used. This device allows constant current electrical stimulations and electromyographic recording of the evoked muscle responses, with integrated impedance feedback. Stimulation consisted of a train-of-five, 1 ms square pulses applied at a frequency of 200 Hz (standard stimulus used in algology experiments) starting at the intensity of 0.5 mA, with 0.2–0.3 mA step size and three direction changes needed before halving/doubling steps. The interstimulation interval was set at 10 s ± 30%. Recording was started 100 ms prior to stimulus onset and lasted until 400 ms thereafter. The NWR threshold was automatically tracked based on the last twelve responses to stimulation, analyzing the poststimulation epoch of 80–250 ms after stimulus onset. The Peak z score criterion was selected, with an evaluation cut off value of 10. Noise was evaluated between 130 and 10 ms before stimulus onset and had to be <15 µV for a recording to be considered valid. Baseline NWR threshold was tracked for a minimum of 15 min before application of the lip twitch. Then the lip twitch was applied for 5 min. The NWR threshold was further continuously determined up to 15 min after twitch removal. The mean baseline NWR threshold was determined for the five minutes preceding the application of the lip twitch and the percent change from baseline during conditioning was quantified. Mean NWR thresholds were also calculated for the intervals 0–5- and 10–15-minutes following twitch removal.

### 2.6 PP/T session

The PP threshold was evaluated using a ProdPro algometer (Top Cat Metrology Ltd, UK). Stimulation was performed through a blunt ended 1 mm diameter pin, pushed against the skin via a pneumatic actuator fixed on the right metacarpus and driven by manually injected air as previously described (16). The actuator was held in place with a boot, and a strap was used to counteract the force generated during stimulation. A dummy actuator, with boot and strap, was applied on the left forelimb at the same height. During stimulation, a constant force rate increase of 2N/s was kept with the guidance of warning LED lights visible on the instrument during operation.

Stimulation was interrupted when a weight shifting to the contralateral limb, a voluntary limb lifting and/or stamping occurred, or when the cut-off force of 25 *N* was reached. At this point, the stimulus was removed, and the peak force (*N*) displayed on the device was recorded as threshold. For stimulations reaching the cut-off, a threshold value of 27.5 *N* was attributed and used for analysis.

Thermal threshold was evaluated on a clipped spot just above the chestnut, on the medial aspect of the antebrachium, using a purpose made hand-held thermode, with a target temperature increase of 0.6°C/sec. The probe (round-shaped, 1 cm diameter) was immediately removed from the skin and the maximal reached temperature recorded as soon as a reaction occurred (see above) or when the cut-off of 52°C was reached. For stimulations reaching the cut-off, a threshold value of 55°C was attributed and used for analysis. Testing sequence (pressure or thermal first) was randomized for each horse and kept constant for consequent measurements. Four threshold measurements for each modality were performed at baseline, with at least 60 s interval between measurements. Thereafter, the lip twitch was applied for five minutes. During the last three minute of application, both thresholds were reevaluated (up to two times each) following the same sequence. Then the twitch was removed, and thresholds were reassessed starting at 5 and 15 min after twitch removal to describe the time course of CPM. At each of these measuring timepoints, thresholds were evaluated 3 times per modality. Whenever more than 2 threshold measurements were obtained for a certain time point, the two closest values were averaged and considered for subsequent statistical analysis.

### 2.7 Sample size calculation

We considered that a 20% threshold difference due to conditioning would indicate a clinically significant CPM. Therefore, for paired T test, a power of 0.8 and alpha 0.05, assuming a SD of 20%, a minimum of 10 horses were deemed necessary. To compensate for a potential drop-out rate of 20% (if horses would show stress or aversion during one of the sessions), 12 horses were included in this experiment.

### 2.8 Statistical analysis

Statistical analysis was performed using Sigma Plot for Windows (Sigma Plot Version 14; Systat Software GmbH, Germany). Descriptive statistics was used for demographic data. Continuous data were checked for normality of distribution using Shapiro-Wilk normality test. Given the non-normal distribution of several variables, data were reported as median [interquartile range (IQR)].

Median and maximal individual stress and aversion scores recorded during the two experimental sessions were compared with the Wilcoxon Signed Rank test. To compare thresholds and physiological values recorded at different time points, the Friedman test with Tukey test for posthoc pairwise analysis was implemented. A *P* value <0.05 was considered statistically significant.

According to recommended standards for reporting of CPM experiments, differences between thresholds recorded before and during conditioning had to be negative to indicate inhibition. Absolute threshold differences (Δ = T_baseline_-T_twitch_) as well as percent changes were determined for the different stimulation modalities. For mechanical and electrical test stimuli, the percent (%) change observed during conditioning compared to baseline was calculated as [(T_twitch_ - T_baseline_)/(T_baseline_)]*100. A threshold modification of at least 20% was considered clinically relevant. Due to the relative nature of the centigrade temperature scale, the calculation of the percent change from baseline for thermal threshold was not performed, as previously suggested (17).

## 3 Results

Twelve healthy horses were included in the study, ten Warmblood (all geldings) and two Freiberger (one gelding and one mare). The median (IQR) age of horses was 8 (5.5–16) year-old and they weighed 570 (535–607) kg, with a body condition score of 3 (3.0–3.4). The ambient temperature during the experimental sessions ranged between 5.1°C and 14.8°C.

Complete data were collected from ten horses in the NWR session and from seven in the PP/T session, respectively. In the first session, ten out of twelve horses completed the experiment, while in the second seven out of ten did. The incomplete data collection leading to the interruption of the experiment was due to severe aversion (score = 4) at the beginning or during application of the twitch in 5 occasions. If this happened during the first experiment, the second did not take place (2 cases, one occurring in the NWR session and one in the PP/T session). Details are presented in Table 2.

**Table 2:** Individual horses included in the study, including their weight, age, sex and breed. For each experimental session it is reported whether the session was completed, interrupted or not performed based on the predefined criteria. Horses for which the first session had to be interrupted, did not participate in the second session (not performed).

Horse	Weight (kg)	Age (years)	Sex	Breed	NWR session	PP/T session
H1	550	16	Gelding	Warmblood	Completed	Interrupted
H2	660	19	Gelding	Warmblood	Interrupted	Not performed
H3	560	7	Gelding	Warmblood	Completed	Completed
H4	600	5	Gelding	Warmblood	Completed	Completed
H5	650	16	Gelding	Warmblood	Completed	Completed
H6	455	3	Gelding	Freiberger	Completed	Completed
H7	570	16	Gelding	Warmblood	Completed	Interrupted
H8	530	7	Gelding	Warmblood	Completed	Completed
H9	455	3	Mare	Freiberger	Completed	Completed
H10	610	9	Gelding	Warmblood	Completed	Interrupted
H11	570	16	Gelding	Warmblood	Completed	Completed
H12	570	7	Gelding	Warmblood	Not performed	Interrupted

Twitch application force was monitored in 9 experimental sessions. The recorded force ranged between 4 and 12 *N*, with a median value of 7.55 *N*. It has to be noticed that force was not constantly applied during twitching, as it was continuously adjusted (slightly released and tensed again) to compensate for the horse's head movements and to avoid slipping, as commonly done in practice.

For the completed sessions, maximal stress scores recorded before and after twitching were always lower than the cut-off. Median stress scores recorded during the 2 stimulation sessions were not statistically different, being 1 (1-1) in both sessions. Aversion scores, attributed every minute during twitch application, varied over time, often reaching a score of 3 (moderate aversion) at one of the observation time points. Median aversion score was 2 (1.75–2.25) during the NWR session and 2 (2-2) during the PP/T session.

### 3.1 NWR session

**Figure 3:**
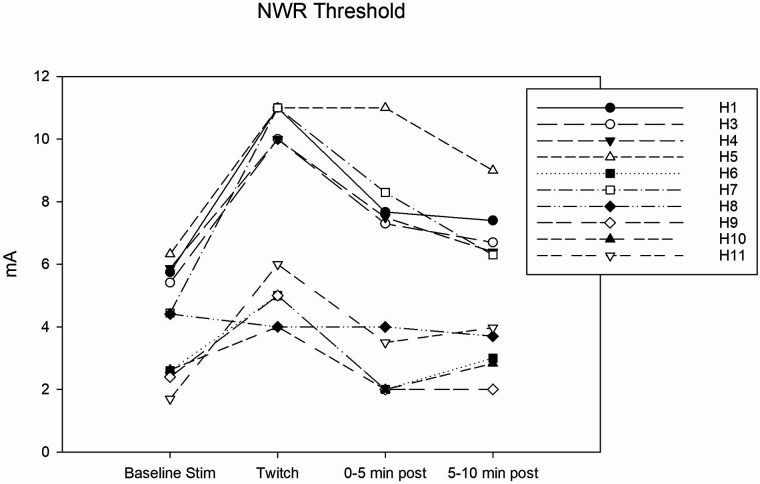
Individual nociceptive withdrawal reflex (NWR) thresholds in mA determined during the following experimental phases: baseline stimulation, twitch, and post twitch (intervals 0–5 min and 5-10 min after twitch removal). All the horses that completed the session are represented.

**Figure 4:**
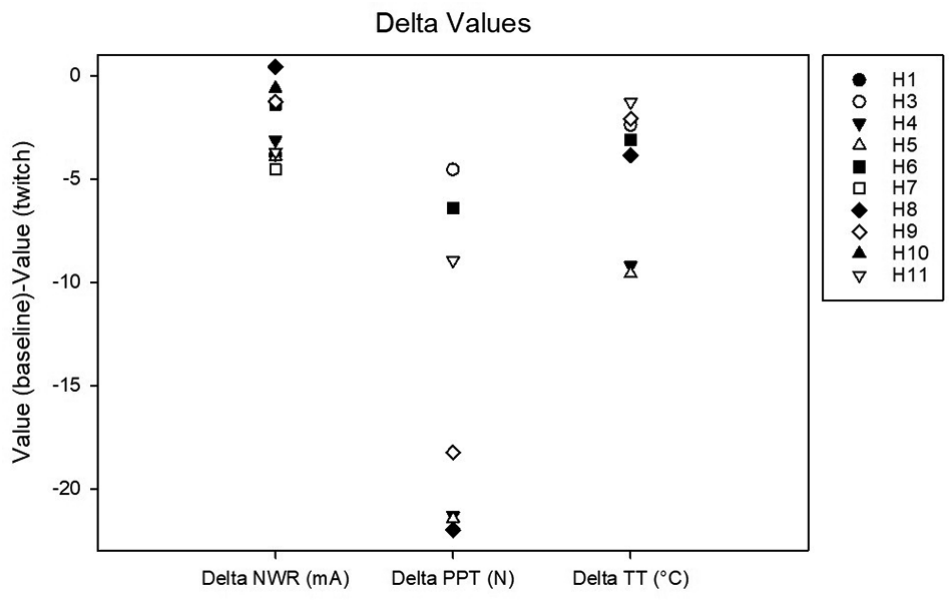
Delta values calculated as the difference between baseline and twitch threshold values for the nociceptive withdrawal reflex (NWR), for pain pressure (PP) and for thermal (T).

Complete data sets were collected for 10 horses. Conditioning significantly affected the NWR threshold (Friedmann test: *P* = 0.002). At baseline, it was 4.4 (2.5–5.8) mA. During twitch application, it raised to a median peak value of 8 (4.7–11) mA (Tukey test: *P* = 0.002) and after removal it decreased to 5.6 (2.0–7.8) mA (interval 0–5 min) and then to 5.1 (2.9–6.9) mA (interval 5–10 min). The peak NWR threshold was reached 4 (2.7–5) minutes after twitch application (Figure 3). The ΔNWR was −2.8 (−3.9 to −1.1) mA. The median percent change during conditioning was 87.9 (65.7–118.2)% (Figure 4).

### 3.2 Pp/T session

**Figure 5:**
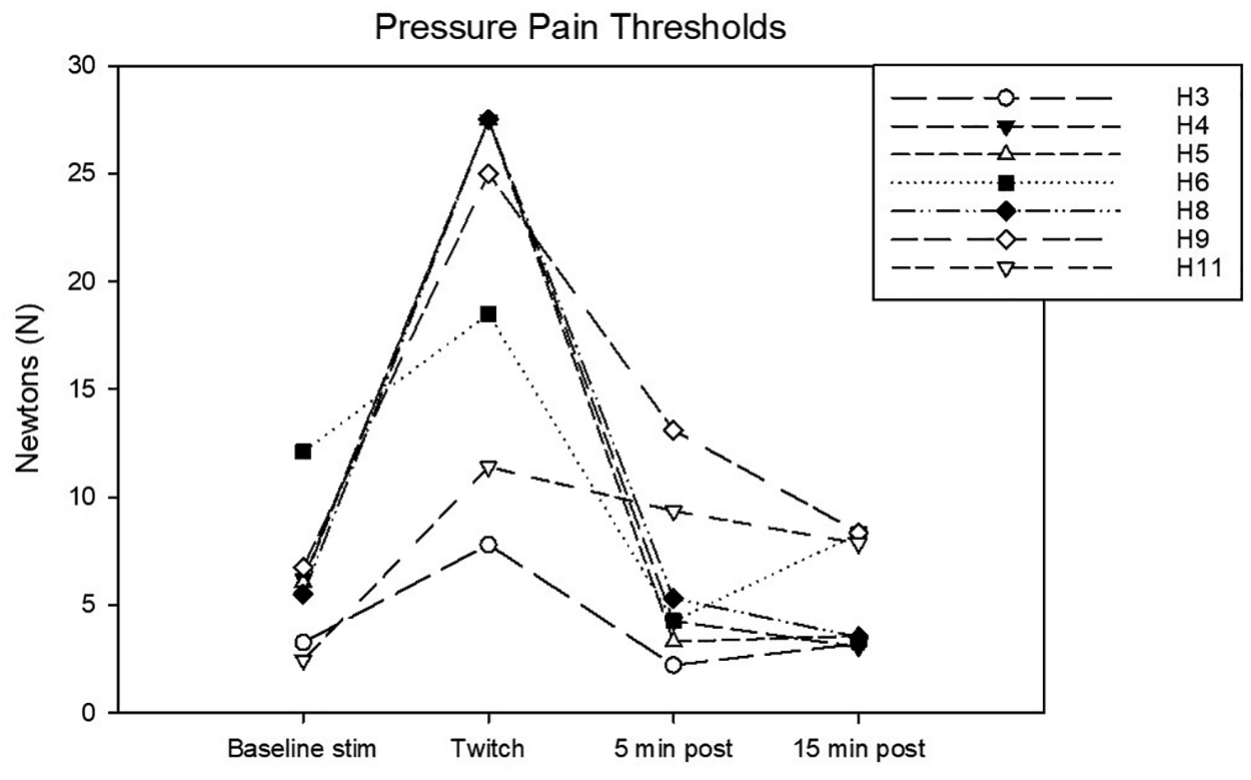
Individual pressure pain (PP) thresholds in N determined during the following experimental phases: baseline stimulation, twitch, and post twitch (intervals 5–10 min and 15–20 min after twitch removal). All the horses that completed the session are represented.

**Figure 6:**
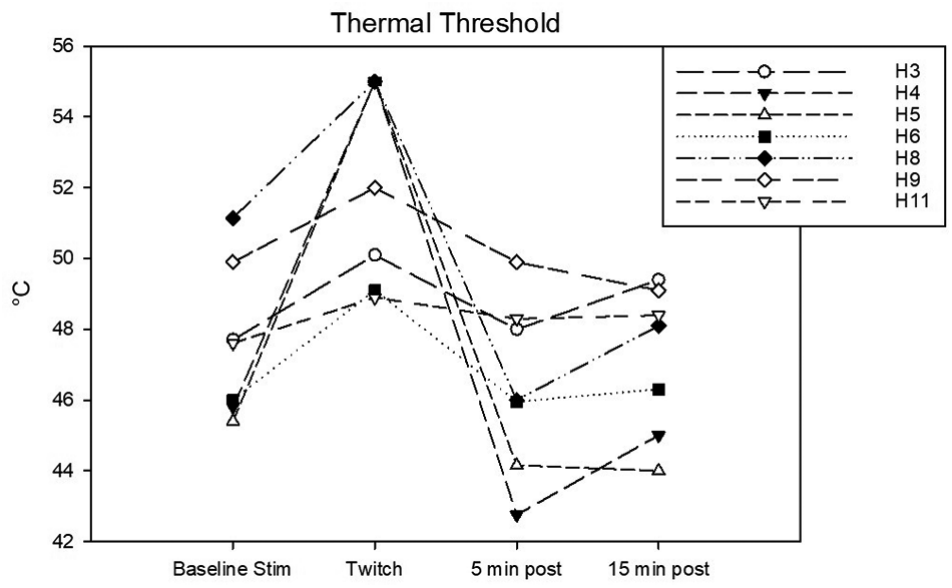
Individual thermal thresholds (T) in °C determined during the following experimental phases: baseline stimulation, twitch, and post twitch (intervals 5–10 min and 15–20 min after twitch removal). All the horses that completed the session are represented.

Complete data sets were collected for 7 horses. Conditioning stimulation significantly affected pressure pain and thermal thresholds (Friedman *P* = 0.003 for both stimulation modalities). At baseline, pressure pain threshold was 6.0 (3.2, 6.7) *N*. During twitch application it raised to 25 (11.4, 27.5) *N* and after removal it decreased to 4.2 (3.3, 9.3) N (interval 5–10 min) and then to 3.5 (3.2, 8.300) *N* (interval 15–20 min) (Tukey *P* = 0.005) (Figure 5). The ΔPPT was −18.2 (−6.4, −21.4) *N*. The median percent change during conditioning was 343.5 (140, 365.3)% (Figure 4). At baseline, thermal threshold was 47.6 (45.8, 49.9)°C. During twitch application it raised to 52 (49.1, 55)°C (Tukey *P* = 0.04) and after removal it decreased to 46 (44.1, 48.3)°C (Tukey *P* = 0.004) and then to 48.1 (45.0, 49.1)°C (Tukey *P* = 0.03) (Figure 6). The ΔT was −3.1 (−9.2, −2.1)°C (Figure 4).

### 3.3 Heart rate and respiratory rate

A statistically significant change in heart rate was observed in both experimental sessions over time (Friedman, *P* < 0.001 for the NWR session and *P* = 0.007 for the PP/T session). Values are reported in Table 2. Overall, median heart rate increased during conditioning compared to baseline and then decreased thereafter. However, only three horses in the NWR session and none in the PP/T session showed >20% increase in heart rate compared to baseline. Two horses in the NWR session (Horse 8 and 9) and three in the PP/T session (Horse 8, 9 and 11) showed a decrease in HR during twitching compared to baseline. Differences between sessions were present only during the baseline stimulation, a higher heart rate being recorded during NWR [39 (34.5–42.1) beats/min] than during PP/T [33.3 (32.6–37.5) beats/min] (Wilcoxon Signed Rank Test *P* = 0.03). A significant change in respiratory rate was observed in both sessions over time (Friedman, *P* < 0.001 for the NWR session and *P* = 0.002 for the PP/T session). Values are reported in Table 2. In both sessions, respiratory rate increased during conditioning and then decreased thereafter. Nine out of 10 horses in the NWR session and all horses in the PP/T session showed >20% increase in respiratory rate. No differences in respiratory rate between sessions were present in any of the experimental phases. Results are reported in Table 3 and figures in Supplementary material (Supplemmentary Figures S2, S3).

**Table 3:** Medians and interquartile ranges for heart rate and respiratory rate for the two sessions (NWR and PP/T) for the following phases: baseline, baseline stimulation, twitch and post-twitch (intervals 0–5 min, 5–10 min after twitch removal).

	Session	Baseline	Baseline Stim	Twitch	0–5 min post	5–10 min post	*P* value
Heart rate (beats/min)	NWR	35.4 (34.4,38.0)	39.1 (34.8,40.9)	41.9 (37.1,43.2)	36.7 (34.8,40.3)	34.2 (32.1,35.8)	<0.001
	PP/T	33.4 (33.3,39.6)	33.4 (32.6,37.5)	36.4 (34.5,37.0)	37.4 (34.6,43.1)	33.1 (30.2,39.7)	0.006
Respiratory rate (breaths/min)	NWR	12 (9.6,14.8)	11.8 (10.0,12.4)	27.3 (15.3,31.7)	14.2 (12,16)	11.8 (10.3,12.8)	<0.001
	PP/T	12 (12,14)	12 (12,18)	28 (16,36)	12 (12,18)	12 (8,20)	0.002

## 4 Discussion

In the current investigation, the utilization of a lip twitch acted as a reliable conditioning stimulus, effectively dampening responses to concurrent nociceptive inputs. This was evidenced by a remarkable elevation in thresholds to electrical, mechanical, and thermal stimulations in the healthy equine subjects under study. Notably, for both electrical and mechanical stimulations, the threshold increase surpassed the hypothesized minimum of 20%, and for thermal stimulations, the observed increase paralleled findings reported following the administration of opioid analgesics in previous studies (18, 19).

The common practice of employing a lip twitch as a method of restraint traces its roots back to ancient Greek and Roman times. Initially, its usage was associated with the observation that horses subjected to twitching tended to exhibit greater tolerance to painful procedures performed on distant body regions, thereby rendering them less hazardous to handle. Already more than two centuries ago, it was hypothesized that pain produced by pressure on the upper lip could diminish the perception and consciousness of pain in other areas (20). In accordance with the results of the present study, other authors reported reduced responses to noxious stimulation during twitching. Lagerveij (21) described a weaker behavioural response to repeated needle pin prick stimulations along the back in horses in presence of the lip twitch and similar observations were described later for donkeys (22). Furthermore, using a semi-quantitative approach, it could be shown that twitching was able to considerably increase thresholds to electrical (23) and thermal stimulations (20) in horses. While weaknesses in reporting and differences in stimulation paradigms do not allow direct data comparison, current evidence and clinical experience unequivocally indicate that twitching evokes a certain degree of antinociception. Interestingly, the physiological mechanism responsible for such effect has been and continues to be the subject of significant scientific debate in equine veterinary research. Among the proposed theories, the “pain inhibits pain” phenomenon, an acupuncture-like effect and stress-induced analgesia are the most mentioned (20). In common, they all assume a central inhibitory effect, potentially also responsible for the sedation and immobilization concomitantly observed. The supposed implication of the endogenous opioid system in its mechanism of action inspired the current study, aiming at testing whether the lip twitch could be used as a conditioning stimulus in a CPM experimental paradigm.

In human studies evaluating CPM, the primary conditioning methods employed include cold pressor pain, which involves immersing the forearm into cold water; noxious heat stimulation, typically administered through a water bath or contact thermode; and the ischemic arm technique, utilizing a tourniquet inflated at a predetermined pressure to induce pain. Among these techniques, the cold pressor pain test consistently demonstrates the most robust and reliable CPM effect (24–26), with the ischemic arm technique following closely behind in terms of efficacy.

In domestic animals, few CPM paradigms have been described. While in calves the ischemic arm technique was deemed adequate to evaluate CPM (27), only continuous mechanical stimulation gave reliable results in dogs (28). Most of the evidence obtained so far suggests that the conditioning stimulus must be noxious to evoke CPM, and that the intensity of pain evoked is associated to the degree of CPM efficacy (12, 29). Furthermore, there seems to be an additive effect of distraction on CPM efficacy, but distraction itself cannot explain the entire CPM effect (30). Interestingly, distraction was also thought to be partly implicated in the lip twitch efficacy in early reports, under the so-called hypothesis of “divertive pain” (21). In human subjects, various stimulation modalities are employed as “test stimuli” to gauge the extent of CPM. These include thermal, mechanical, chemical, and electrical stimuli, each utilized with phasic, tonic, or summation approaches. Noxious stimuli can be administered either to a remote location or to the same body part as the one undergoing conditioning stimulation. Additionally, the tissues targeted for stimulation may be superficial (e.g., skin) or deep (e.g., muscles, viscera), providing a comprehensive assessment of pain modulation mechanisms (12). In the context of the present study, three distinct stimulation modalities were applied across two experimental sessions. The adoption of multiple test stimuli within the same experimental setup has been recommended to enhance mechanistic comprehension and confirm test validity (31).

During one session, electrical stimulation was employed to elicit the NWR using a continuous automated threshold tracking device. This device relies on a quantifiable neurophysiological outcome, specifically electromyographic activity recorded within a defined post-stimulation time epoch, to establish and modify the input, namely the stimulation intensity.

The methodology employed in the current study builds upon several previous reports that have delineated the NWR and its pharmacological modulation in horses. Furthermore, the NWR model has been used in humans to assess CPM (32, 33). The utilization of continuous tracking, as opposed to a singular threshold definition, permits the ongoing assessment of treatment efficacy or procedural effects in a continuous manner, thereby facilitating a more precise determination of onset and duration of action. Given that the threshold determination process was automated and continuous, with randomized yet brief interstimulation intervals, it would have posed a risk of interference to combine this stimulation modality with another. Consequently, the other two modalities, thermal and mechanical, were administered in a distinct session to ensure data integrity and prevent potential confounding factors. In the PP/T session, an interstimulation interval of at least 60 s was adhered to between two consecutive stimuli. Additionally, stimulation was avoided during the initial 2 min following twitch application. This measure was implemented based on findings from previous reports, which suggested that a certain time is necessary for the onset of twitch action to manifest effectively. While thermal testing was performed with a hand-held device, the mechanical was based on a fixed-mounted design. Advantage of this last one was that no additional contact with the horse was necessary during the experiment. Conversely, in the case of thermal stimulation, prompt removal of the thermode upon reaching the threshold was practiced. This approach served to mitigate the potential risk of sensitization, as cooling of the sensor may occur with a slight delay even after the stimulus is discontinued. Given that various stimulation modalities exhibit differential responses to analgesic agents, the concurrent use of multiple tests aimed to investigate whether twitching exerts a specific inhibitory effect on particular afferent inputs, thus offering deeper insights into its mechanisms of action. For instance, electrically induced NWR has demonstrated heightened sensitivity to alpha-2 agonists and local anesthetics, whereas its responsiveness to opioids is comparatively reduced. Conversely, the sensitivity of thermal stimuli applied at slow increasing rates is known to be diminished by opioids. Considering the observed effects across all three modalities, it is reasonable to infer that both opioid and non-opioid mechanisms of endogenous pain inhibition were activated by the lip twitch in the horses of the present study.

The contribution of the endogenous opioid system in the mechanism of action of the lip twitch has been substantiated by several studies. Most of the reports indicate an early (21–23) or even immediate (20) rise in β-endorphin after application, followed by a continuous rise with a peak occurring at around 5 min and a tendence to decline thereafter. Such a decline was hypothesized to explain the biphasic effect observed in case of prolonged twitch application, with sedation observed in the first 5 min followed by restlessness, aversive behaviour and high sympathetic tone thereafter (34). In Lagerweji (21), the observed increased in β-endorphin levels were interpreted as a demonstration that twitching acts as acupuncture in inducing sedation and analgesia. However, a large body of evidence has shown that in equines β-endorphines are released during early stages of stress (35) and in acute painful conditions such as during colic (36). Its precursor, proopiomelanocortin (POMC) is produced in the anterior pituitary in response to increasing levels of hypothalamic corticotropin releasing hormone (CRH). In presence of a stressor, the activation of the autonomic nervous system and hypothalamic pituitary adrenal (HPA) axis promotes the secretion of circulating catecholamines, β-endorphines, adrenocorticotropic hormone (ACTH), and cortisol. All these substances have been abundantly used to monitor animal welfare and emotional responses to stressors in the literature (35). In equines, β-endorphin release appears to occur early during stressful events. Through a negative feedback mechanism, it inhibits the secretion of CRH, suggesting a role in modulating the stress cascade. Furthermore, this release pattern may facilitate active coping strategies and mitigate pain, as previously shown (35, 36). In the present study, stress hormones were not measured, thus their potential correlation with the observed antinociceptive effects cannot be directly investigated.

On the contrary, heart rate and respiratory rate were measured before, during and after twitching. While heart rate and heart rate variability have been monitored in several other twitch studies (20–22, 34, 37, 38), previous data about respiratory rates could not be found in the literature. Our heart rate findings overlap with those previously reported by some authors (20, 38). We observed individual variations in the heart rate response to twitch, with some horses increasing and other decreasing frequency. Overall, the extent of variation was rather low (always lower than 20%), and the same horses which responded with bradycardia to the first challenge did the same at the second occasion, indicating that there is a rather individual predisposition for the direction of response. As most of the horses displayed bradycardia in response to twitch in other studies (21, 34, 37), a trigemino-vagal reflex with a shift toward parasympathetic dominance was hypothesized and associated to the analgesic and sedative effect observed. On the contrary, in a study in donkeys, heart rate and heart rate variability data indicated increased sympathetic tone in these animals, which also showed aversive behaviour (22). Thus, the present and past evidence regarding twitch effects on heart rate is rather inconclusive and points toward individual differences and external factors that might induce a shift of the autonomic balance toward either the sympathetic or parasympathetic dominance. Further work on the collected heart rate variability data, not included in the current report, might contribute to a better understanding of this phenomenon.

Differently from what observed for heart rate, an impressive, clinically relevant rise in respiratory rate was consistently observed during the twitch application in all the horses of the present study. Interestingly, as soon as the twitch was removed, this parameter immediately normalized. Release of plasma catecholamines, adrenaline, noradrenaline and dopamine, classically accompanies the activation of the sympathetic adrenomedullary system, which reflects the most immediate, but also generally short lasting, response to stress. Catecholamines relax bronchioles and increase ventilation, thus preparing the organism for a flight and fight reaction (39). In a study in horses, adrenalin and noradrenalin increased significantly after twitch application, with adrenalin levels remaining higher than noradrenalin longer after removal. Hematocrit increased quickly after application too, indicating that twitching acts as a potent stressor, while cortisol increase was delayed, as expected due to its physiological function and release pattern (20). These observations would substantiate the hypothesis that the effects of the twitch, commonly targeted in clinical practice, are at least partially mediated by a stress-related sympathetic activation, one of the mechanisms known to be implicated in endogenous pain inhibition (40). Whether stress is rather related to pain, thus to a physiologically mediated event or to forceful restraint, thus rather an emotional challenge, or both cannot be distinguished at present. Further investigations, including both physical challenges and emotional stressors, such as social isolation, confinement in unfamiliar environments, and novel object tests (41), are necessary to explore how these factors differently affect endogenous pain modulation in horses.Concerning behaviour, the literature reveals a wide spectrum of possible reactions to the lip twitch, spanning from deep sedation to freezing, clear aversion to handling, escape behaviour, or even dangerous fight attitudes. Most reports described sedative effects or even lethargy in a high proportion of subjects. Interestingly, the administration of naloxone reversed sedation, leading to an increase occurrence of aversive behaviour (20, 21). These findings suggest a potential association between sedation and the levels of circulating b-endorphins or other endogenous opioids. In the study by Schelp (20) and in a study involving donkeys (22), it was highlighted that not all subjects displayed sedation following twitching. However, antinociception was still evident, suggesting that the lip twitch might activate both opioidergic and non-opioidergic mechanisms of endogenous pain inhibition.

In the current study, a notably high occurrence of aversive behaviour was observed, surpassing what is typically encountered in a mixed population of equine patients treated in field or clinical conditions. This heightened prevalence could potentially be attributed to the specific population of horses included in the study, all of which were selected for demanding public tasks within the armed forces. It is plausible that their personality traits are distinct and predispose them to more proactive reactions during forced restraint. However, this hypothesis would require further investigation to be confirmed. Furthermore, the test stimuli applied before twitching to obtain baseline thresholds might have caused a subliminal arousal state, that might have modified subsequent behavioural reactions to the conditioning. This hypothesis might be at least partly confirmed by the observation that in the NWR session, the heart rate during baseline stimulation was significantly higher than during baseline in absence of stimulation. Future specific HRV investigations on the presented HR data will possibly contribute to define the role of the autonomic balance in the twitch action and in endogenous pain modulation mechanisms in horses.

In summary, while several factors such as individual predisposition, past experiences, environment, and coexisting stressors could contribute to determine the behavioural response, predicting a specific pattern appears unrealistic at present. The complexity and variability of equine behaviour underscores the need for further research to better understand the dynamics at play in response to the lip twitch.

In the current study, it was observed that some horses exhibited sensitization to the lip twitch. It is highly probable that many of these horses had previous experiences with this restrain method. This likelihood could explain the exclusion of certain horses during the first experimental session and, most likely, their subsequent exclusion during the second session as well. Research has indicated that sensitization to aversive events can occur in horses after relatively few exposures and such sensitization can persist for extended periods of time (42). While it would certainly be interesting to address this issue specifically in future investigations, it can be deduced that for certain subjects, lip twitching constituted a stressful, potentially fear-inducing, and aversive event.

The current study has several limitations. First, horses needed to be retrained to perform the testing, and this might have induced a basic level of stress influencing the whole procedure and potentially the CPM results. Future studies will need to compare antinociceptive extent of conditioning with and without restrain to evaluate whether this factor has a relevant influence. To this end, different conditioning and test stimulations modalities will need to be used, as the ones adopted in the present study cannot be applied in freely moving animals. Thus, this limitation is inherent to the lip twitching, which must be performed by a handler holding it continuously as conditioning stimulus. Second, the limited number of subjects included, and in particular of females, precludes thoroughly investigating the role of influencing factors on the different behavioural and physiological response patterns observed. This would need a bigger sample size to provide credible results. Third, as mentioned above, plasmatic concentrations of stress hormones, catecholamines and endogenous opioids were not measured. Blood sampling implies an additional handling, and an increased severity level, even if performed through an indwelling catheter. As the focus of the current investigation was on antinociception, it was decided to minimize sources of distraction and the number of interventions needed around the experiment.

In conclusion, based on the current findings, the question of whether lip twitching should be utilized as a conditioning method in CPM studies appears debatable. On one hand, if conditioning is to effectively induce pain, aversive behavioural signs must be inherently tolerated to some extent; on the other, it is imperative to establish stringent cut-off criteria to prevent exposing sensitive individuals to elevated stress and excluding them from testing. Similar considerations should apply to the general use of the lip twitch as a restrain technique in horses.

## Data Availability

The original contributions presented in the study are included in the article/Supplementary Material, further inquiries can be directed to the corresponding authors.
