# Supplementary material for: Investigating conditioned pain modulation in horses: can the lip-twitch be used as a conditioning stimulus?
Source: Front Pain Res (Lausanne). 2024 Oct 24;5:1463688. doi: 10.3389/fpain.2024.1463688 (PMC11540819; doi:10.3389/fpain.2024.1463688)
Supplement: Supplementary file 1 [file Table1.docx]

Supplementary Material

# Supplementary Figures

**
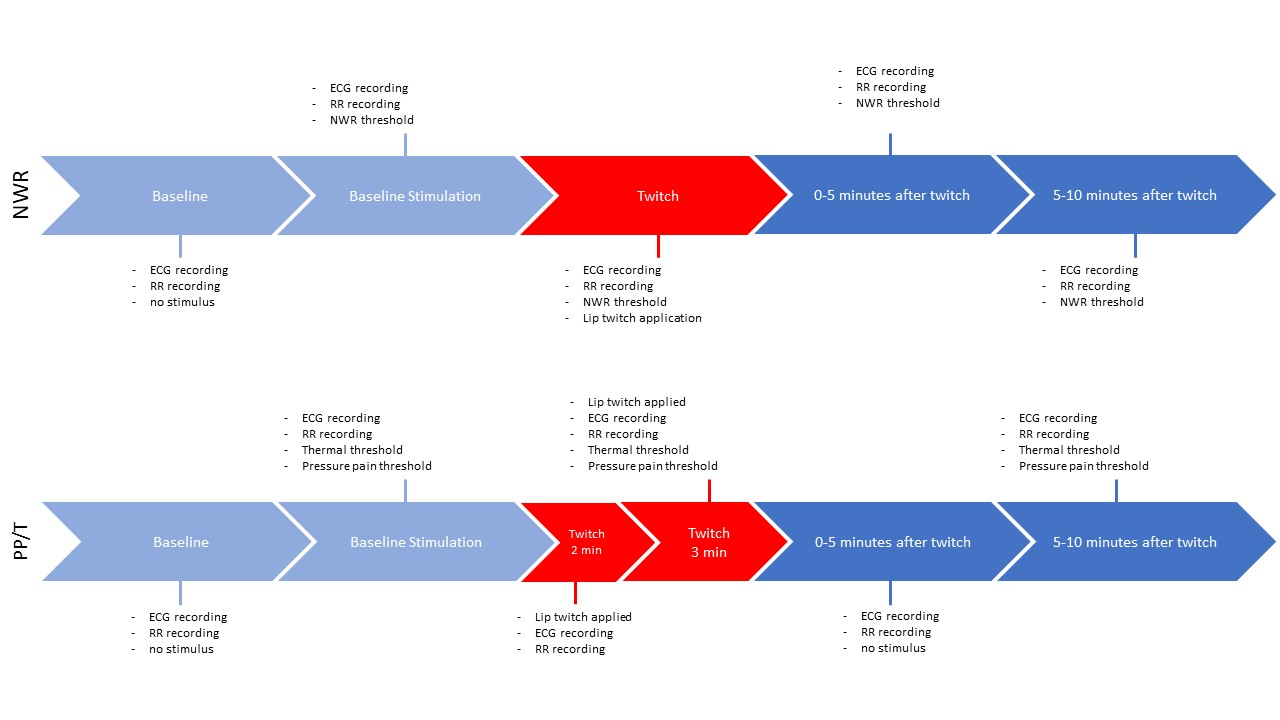
**

**Supplementary Figure 1.** Timeline of the experiment. The two experimental sessions are represented. The nociceptive withdrawal reflex (NWR) session can be viewed on the top half of the figure and pain pressure/thermal (PP/T) located on the bottom half. The phases are baseline: no stimulation occurred; baseline stimulation, where either electrical (NWR) or mechanical/thermal (PP/T) stimulation occurred; twitch, where the lip-twitch was applied; then the post-twitch phases divided into 5-minute intervals. Recordings and stimulations performed are reported within the figure.

**
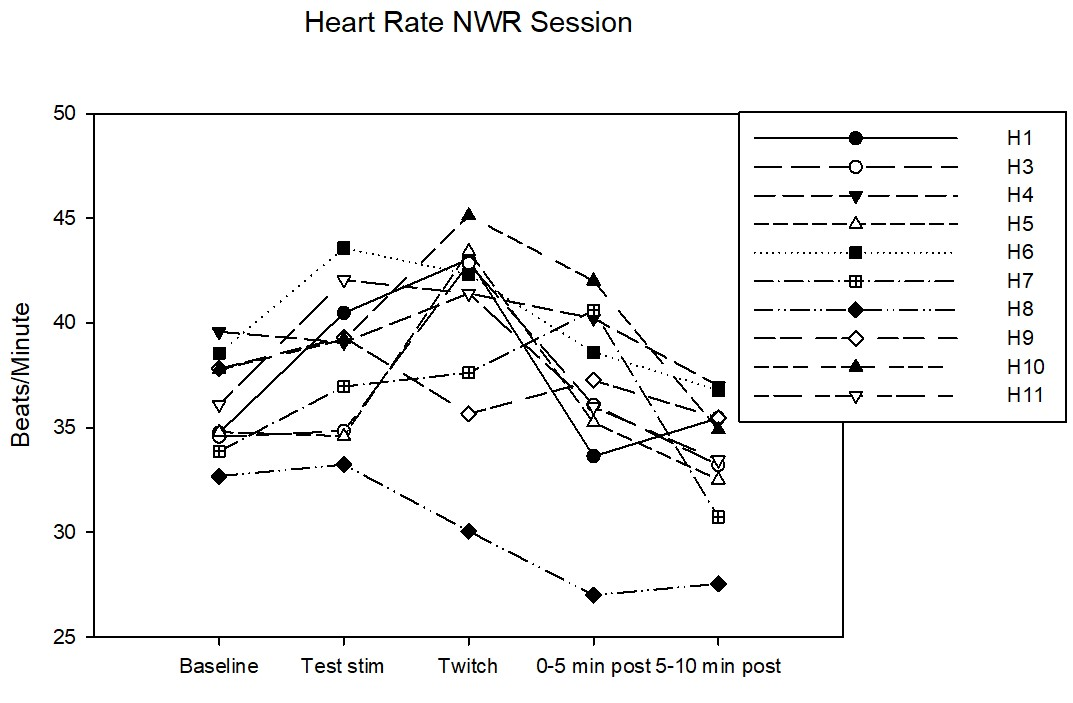
**A.


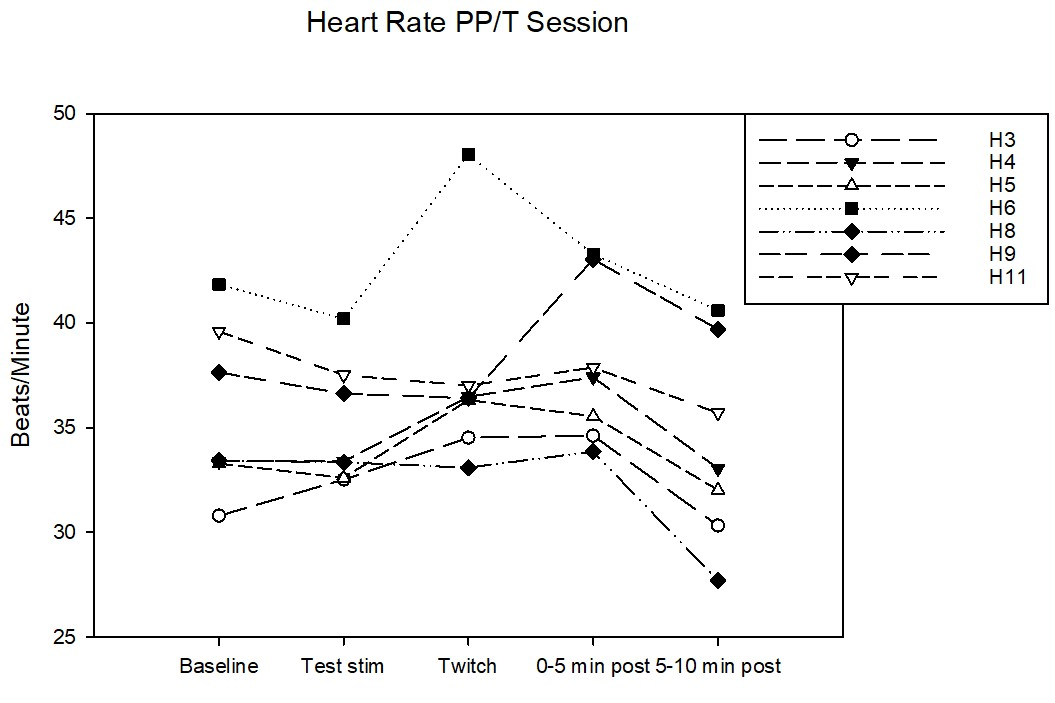
B.

**Supplementary Figure 2.** A) Individual average heart rate during the NWR session calculated for the following experimental phases: baseline, baseline stimulation (Test stim), twitch and post-twitch (intervals 0-5 mins and 5-10 mins after twitch removal). B) Individual average heart rate during the PP/T session calculated for the following experimental phases: baseline, baseline stimulation (Test stim), twitch and post-twitch (intervals 0-5 mins and 5-10 mins after twitch removal).


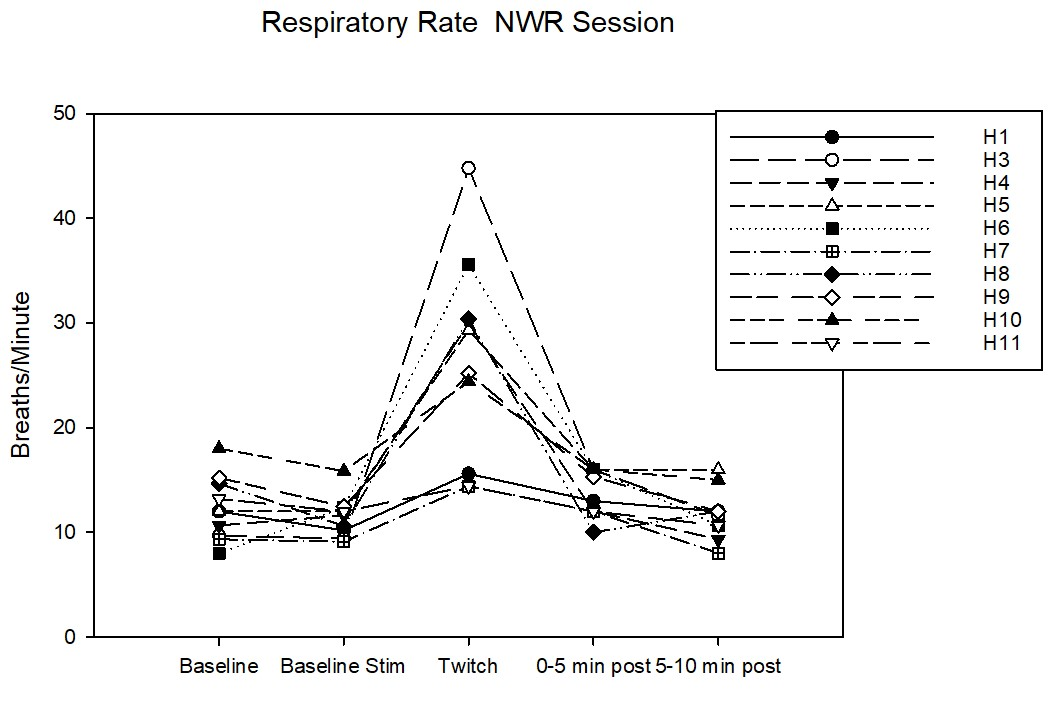
A.


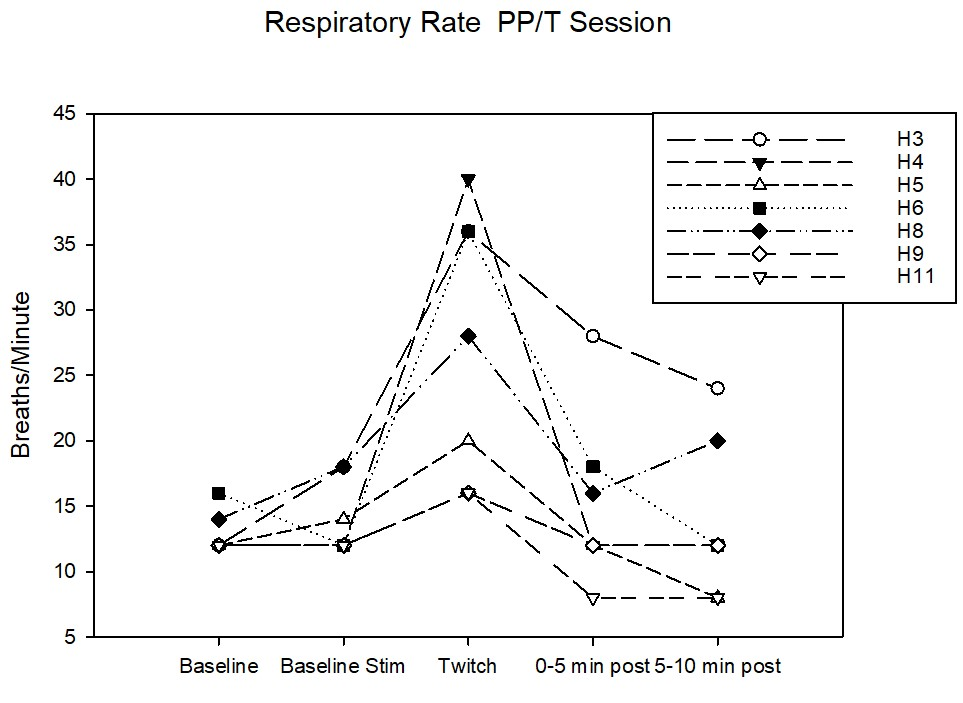
B.

**Supplementary Figure 3.** A) Individual average respiratory rate during the NWR session calculated for the following experimental phases: baseline, baseline stimulation (Test stim), twitch and post-twitch (intervals 0-5 mins and 5-10 mins after twitch removal). B) Individual average respiratory rate during the PP/T session calculated for the following experimental phases: baseline, baseline stimulation (Test stim), twitch and post-twitch (intervals 0-5 mins and 5-10 mins after twitch removal).
